# Supplementary material for: Exploring the Role of Miniemulsion Nanodroplet Confinement on the Crystallization of MoO3: Morphology Control and Insight on Crystal Formation by In Situ Time-Resolved SAXS/WAXS
Source: Nanomaterials (Basel). 2023 Mar 14;13(6):1046. doi: 10.3390/nano13061046 (PMC10051643; doi:10.3390/nano13061046)
Supplement: Supplementary file 1 [file nanomaterials-13-01046-s001.zip › nanomaterials-2264354-supplementary.pdf]

Supporting Information

Exploring the role of miniemulsion nanodroplets confinement on the crystallization of MoO_3: morphology control and insight on crystal formation by *in situ* time-resolved SAXS/WAXS

Francesca Tajoli [1], Maria Vittoria Massagrande [1], Rafael Muñoz-Espí [2] and Silvia Gross [1,3,*]

[1] Dipartimento di Scienze Chimiche (DiSC), Università degli Studi di Padova, Via Marzolo 1, 35131 Padova, Italy
[2] Institute of Materials Science (ICMUV), Universitat de València, Catedràtic José Beltrán 2, 46980 Paterna, València, Spain
[3] Karlsruher Institut für Technologie (KIT), Institut für Technische Chemie und Polymerchemie (ITCP), Engesserstrasse 20, 76131, Karlsruhe, Germany
* Correspondence: silvia.gross@unipd.it

1. Samples lists

Miniemulsion samples are listed in Table S1, together with relative experimental conditions.

Table S1. Molybdenum oxide samples synthesized by ME approach.

Sample name	Conc of AHM solution (M)	AHM:HNO_3 molar ratio	US after acid addition	Reaction time
MO01-me	0.10	1:10	yes	24 h
MO02-me	0.15	1:10	yes	24 h
MO03-me	0.20	1:10	yes	24 h
MO04-me	0.25	1:10	yes	24 h
MO05-me	0.20	1:15	yes	24 h
MO06-me	0.20	1:20	yes	24 h
MO07-me	0.20	1:25	yes	24 h
MO08-me	0.20	1:10	no	24 h
MO09-me	0.20	1:20	no	24 h
MO10-me	0.20	1:20	no	48 h
MO11-me	0.20	1:10	yes	5 min
MO12-me	0.20	1:10	yes	6 h
MO13-me	0.20	1:10	yes	18 h
MO14-me	0.20	1:10	no	5 min
MO15-me	0.20	1:10	no	6 h
MO16-me	0.20	1:10	no	18 h

Batch samples are listed in Table S2, together with relative experimental conditions.

Table S2. Molybdenum oxide samples synthesized by batch approach.

Sample name	Conc of AHM solution (M)	AHM:HNO_3 molar ratio	US after acid addition	Reaction time
MO01-b	0.10	1:10	no	24 h
MO02-b	0.15	1:10	no	24 h
MO03-b	0.20	1:10	no	24 h
MO04-b	0.25	1:10	no	24 h
MO05-b	0.20	1:15	no	24 h
MO06-b	0.20	1:20	no	24 h
MO07-b	0.20	1:25	no	24 h
MO08-b	0.20	1:10	yes	24 h
MO09-b	0.20	1:10	no	5 min
MO10-b	0.20	1:10	no	6 h
MO11-b	0.20	1:10	no	18 h

2. ATR spectra of miniemulsion and batch samples

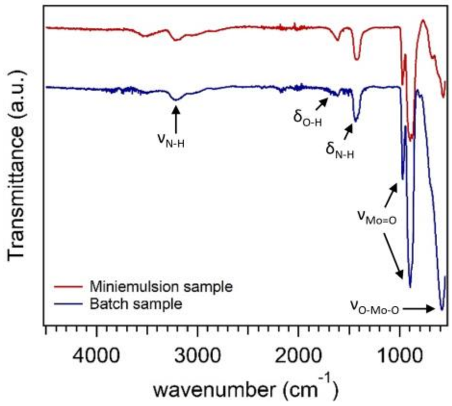

Figure S1. ATR spectra of miniemulsion (red) and batch (blue) samples.

3. Effect of AHM concentration

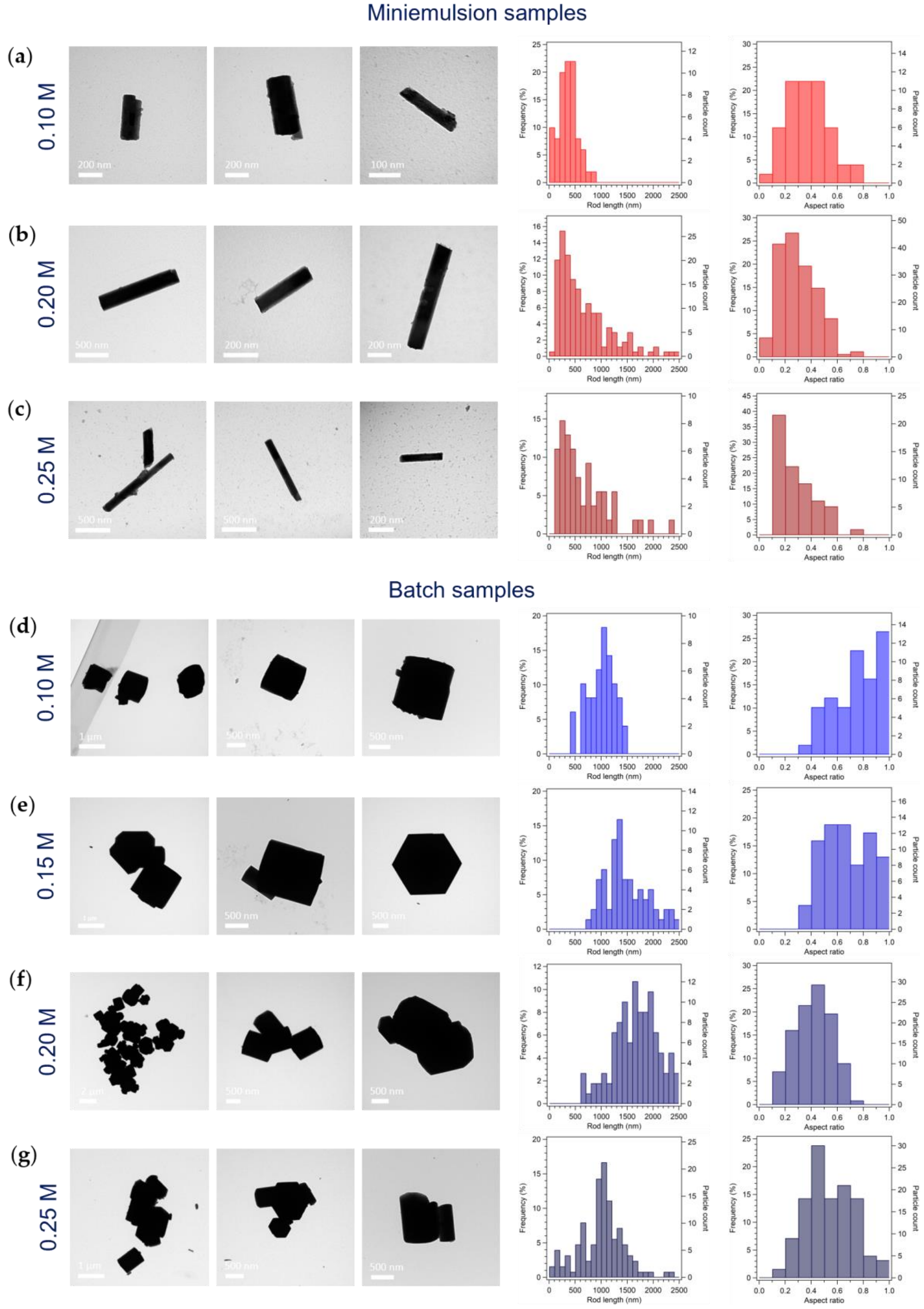

Figure S2. TEM micrographs and relative histograms of rod length and aspect ratio distributions of (**a-c**) miniemulsion and (**d-g**) batch samples synthesized at different AHM concentrations (0.10, 0.15, 0.20, and 0.25 M) and constant AHM: HNO_3 molar ratio (1:10 mol) and reaction time (24 h). *ME sample 0.15 M not reported because of bad quality of TEM micrographs.

Table S3. Average rod length, width and aspect ratio (AR=width/length) and standard deviations of miniemulsion and batch samples synthesized at different AHM concentrations (0.10, 0.15, 0.20, and 0.25 M) and constant AHM: HNO_3 molar ratio (1:10 mol) and reaction time (24 h).

	Sample name	[AHM] (M)	Length (nm)	Width (nm)	Aspect ratio
ME	MO01-me	0.10	355 ± 180	125 ± 70	0.4 ± 0.1
	MO02-me [1]	0.15	565 ± 271 [1]	237 ± 88 [1]	0.4 ± 0.2 [1]
	MO03-me	0.20	656 ± 499	176 ± 152	0.3 ± 0.1
	MO04-me	0.25	659 ± 486	159 ± 134	0.3 ± 0.1
batch	MO01-b	0.10	998 ± 259	759 ± 292	0.7 ± 0.2
	MO02-b	0.15	1488 ± 429	1005 ± 418	0.7 ± 0.2
	MO03-b	0.20	1697 ± 445	730 ± 345	0.4 ± 0.1
	MO04-b	0.25	1108 ± 419	576 ± 340	0.5 ± 0.2

[1] Insufficient statistics due to bad quality of TEM micrographs

4. Effect of AHM:HNO_3 molar ratio

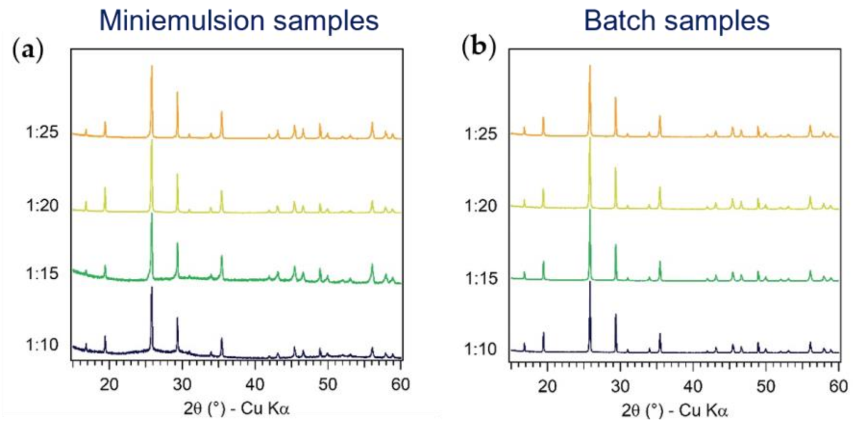

Figure S3. Comparison of XRD pattern of h-MoO_3 samples synthesized in (**a**) miniemulsion and (**b**) batch as a function of AHM: HNO_3 molar ratio (1:10, 1:15, 1:20, 1:25 mol), keeping constant the AHM concentration (0.20 M) and the reaction time (24 h).

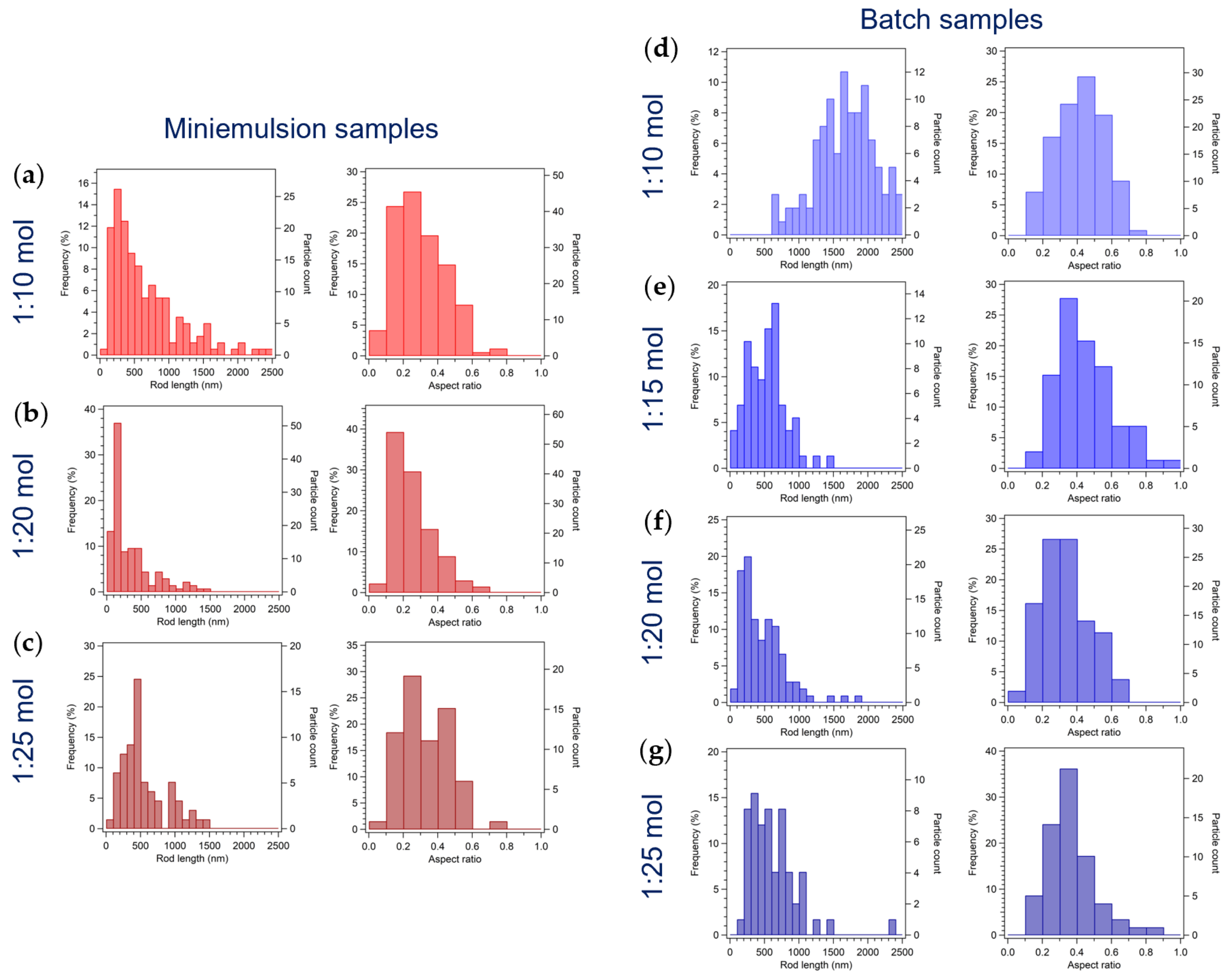

Figure S4. Histograms of rod length and aspect ratio distributions of (**a-c**) miniemulsion and (**d-g**) batch samples synthesized at different AHM: HNO_3 molar ratio (1:10, 1:15, 1:20, and 1:25 mol) and constant AHM concentrations (0.20 M) and reaction time (24 h). *ME sample 1:15 mol not reported because of bad quality of TEM micrographs.

Table S4. Average rod length, width and aspect ratio (AR=width/length) and standard deviations of miniemulsion and batch samples synthesized at different AHM: HNO_3 molar ratio (1:10, 1:15, 1:20, and 1:25 mol) and constant AHM concentrations (0.20 M) and reaction time (24 h).

	Sample name	AHM:HNO_3 mol	Length (nm)	Width (nm)	Aspect ratio
ME	MO03-me	1:10	656 ± 499	176 ± 152	0.3 ± 0.1
	MO05-me [1]	1:15	-	-	-
	MO06-me	1:20	358 ± 371	94 ± 107	0.3 ± 0.1
	MO07-me	1:25	544 ± 327	177 ± 134	0.3 ± 0.1
batch	MO03-b	1:10	1697 ± 445	730 ± 345	0.4 ± 0.1
	MO05-b	1:15	524 ± 280	248 ± 213	0.5 ± 0.2
	MO06-b	1:20	466 ± 322	143 ± 108	0.3 ± 0.1
	MO07-b	1:25	618 ± 365	216 ± 128	0.4 ± 0.1

[1] Insufficient statistics due to bad quality of TEM micrographs

5. Effect of ultrasounds and reaction time

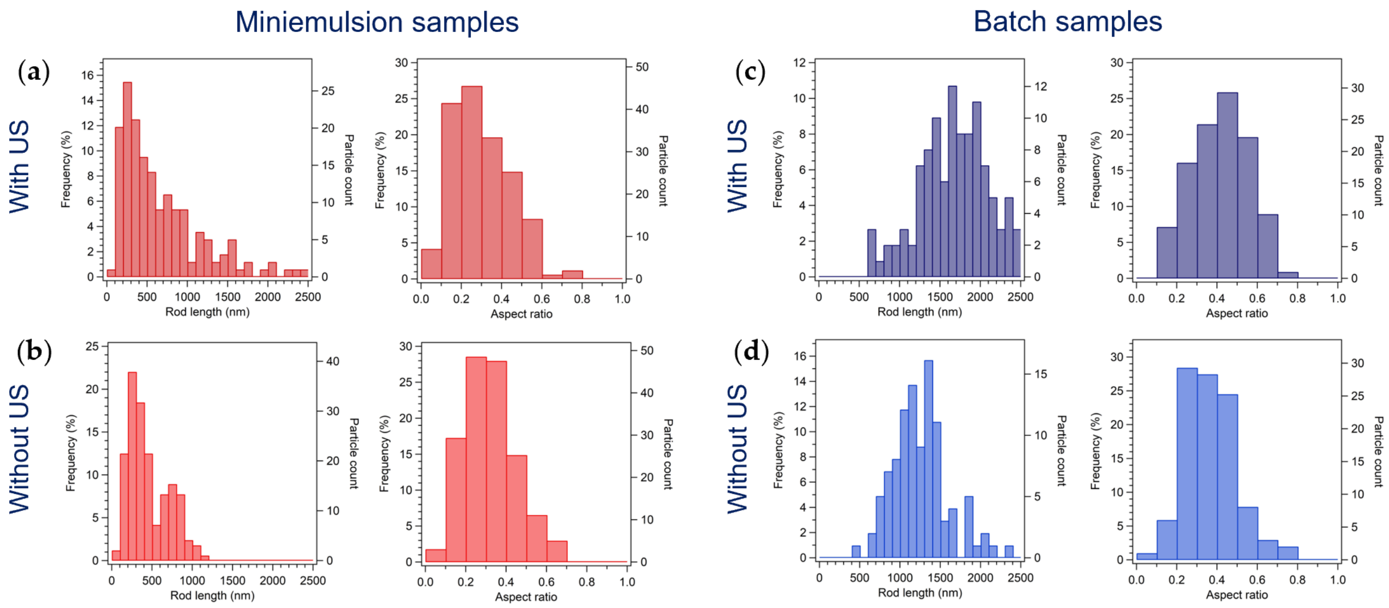

Figure S5. Histograms representing rod length and aspect ratio distributions of (**a**,**b**) miniemulsion and (**c**,**d**) batch samples synthesized by (**a**,**c**) applying or (**b**,**d**) not applying US after acid addition. [AHM] = 0.20 M; AHM: HNO_3 1:10 mol, 24 h.

Table S5. Average rod length, width and aspect ratio (AR=width/length) and standard deviations of miniemulsion and batch samples synthesized with 18 and 24 h of reaction time, by applying or not US after acid addition and with constant AHM concentrations (0.20 M) and AHM: HNO_3 molar ratio (1:10 mol).

	Sample name	Reaction time (h)	US after acid add.	Length (nm)	Width (nm)	Aspect ratio
ME	MO13-me	18	Yes	463 ± 265	79 ± 56	0.2 ± 0.1
	MO03-me	24	Yes	656 ± 499	177 ± 152	0.3 ± 0.1
	MO09-me	24	-	450 ± 248	140 ± 99	0.2 ± 0.1
batch	MO11-b	18	-	1058 ± 308	547 ± 294	0.5 ± 0.2
	MO03-b	24	-	1697 ± 445	730 ± 345	0.4 ± 0.1
	MO08-b	24	Yes	1248 ± 345	470 ± 234	0.4 ± 0.1

[1] Insufficient statistics due to bad quality of TEM micrographs

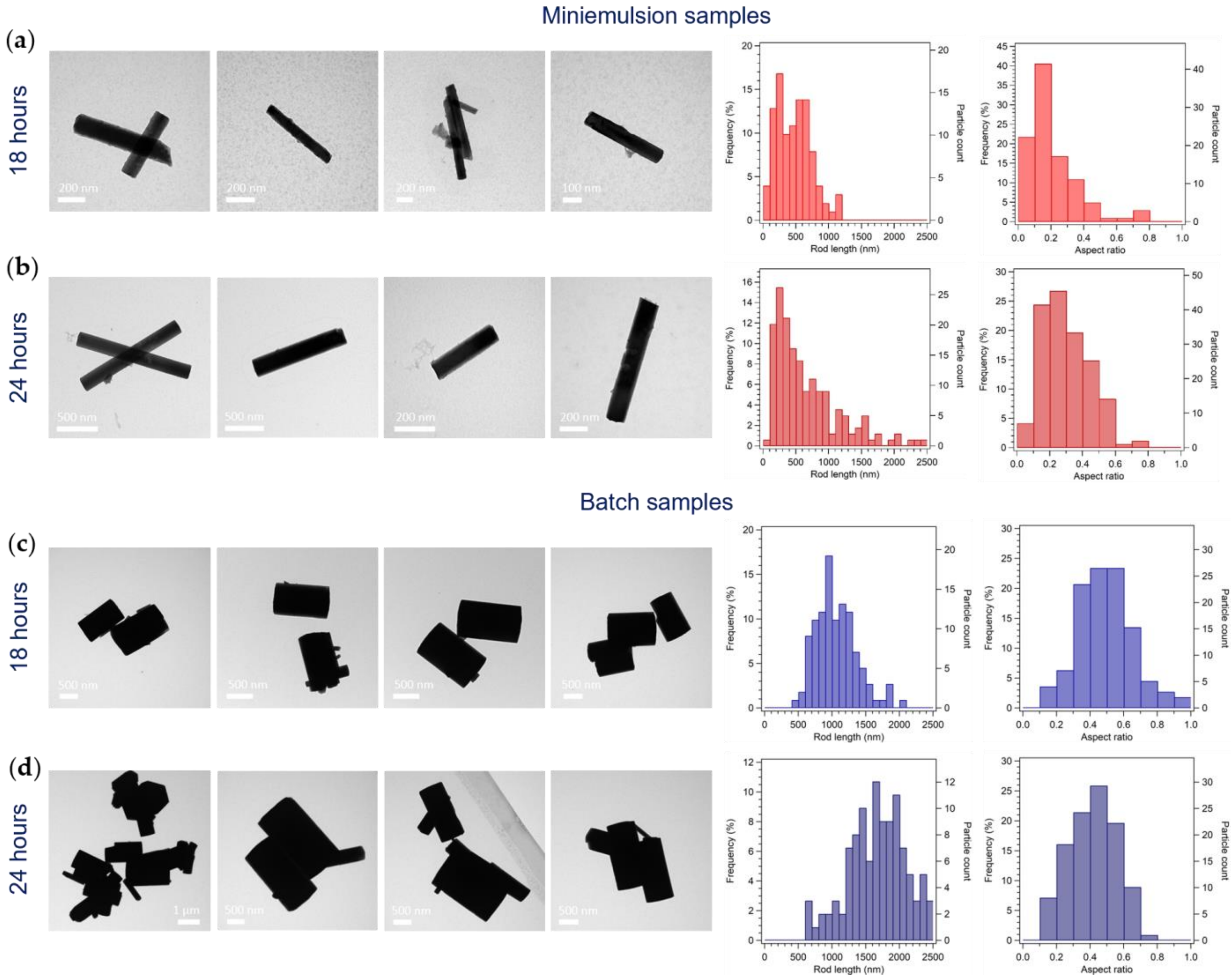

Figure S6. TEM micrographs and relative histograms of rod length and aspect ratio distributions of (**a,b**) miniemulsion and (**c,d**) batch samples synthesized with reaction times of 18 hours and 24 hours [AHM] = 0.20 M; AHM: HNO_3 1:10 mol.

6. *In situ* time-resolved SAXS/WAXS study

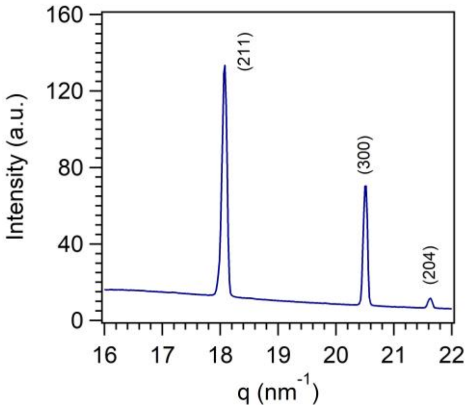

Figure S7. WAXS profile of a batch sample synthesized with AHM: HNO_3 of 1:10 mol at the SAXS beamline at Elettra Sincrotrone Trieste and measured after 24 h.

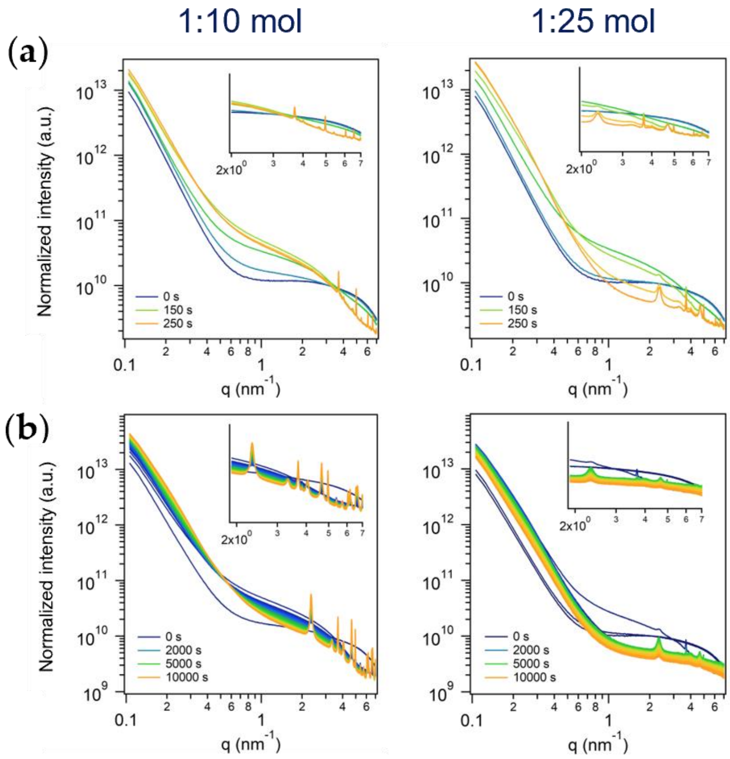

Figure S8. *In situ* time-resolved log-log plots of SAXS patterns of the series of experiments performed in ME employing PGPR as surfactant and with different AHM:HNO_3 molar ratios. (**a**) A SAXS pattern every 50 s from the start of acid addition (t = 0 s) to the end of US (step iii) (t = 250 s) and (**b**) a SAXS pattern every 100 s from the start of acid addition (t = 0 s) to 10000 s. Insets: zoom on Bragg peaks (1.9-7 nm^{-1}).
